# Plaque verruqueuse brunâtre rétroauriculaire

**DOI:** 10.11604/pamj.2014.18.265.5000

**Published:** 2014-07-31

**Authors:** Ahlam Abdou, Mohammed Ait Ourhroui

**Affiliations:** 1 Service de Dermatologie, CHU Ibn Sina, Université Med V, Souissi, Rabat, Maroc

**Keywords:** Plaque verruqueuse, naevus sébacé de Jadassohn, hamartome cutanée, warty plaque, nevus sebaceous of Jadassohn, cutaneous hamartoma

## Image en medicine

Naevus sébacé de Jadassohn est un hamartome cutanée congénitale avec une incidence estimée de 0,3% chez les nouveau-nés. Il est généralement situé sur la tête et du cou. Il s'agit le plus souvent d'une lésion solitaire ovalaire de petite taille 2 a 3 cm. Elle apparait à la naissance sous forme de plaque rosée granitée du cuir chevelu ou du front. A la puberté, la lésion devient mamelonnée et pigmentée puis elle devient ferme et kératosique. Le diagnostic différentiel est représenté par la kératose séborrhéique et les verrues. L'examen histologique montre une hyperplasie épidermique, une papillomatose, avec hyperkératose et un grand nombre de glandes sébacées matures agglomérées des follicules pileux avortés et des glandes apocrines ectopiques. L'étude menée par Merrot et al a permis de mettre en évidence que les hamartomes de jadassohn sont le lit de tumeurs basaloides, les trichoblastomes et que la transformation en carcinome bascellulaire était beaucoup moins fréquente que ce qui était avancé auparavant. -Le trichoblastome est une tumeur pilaire bénigne souvent confondue histologiquement avec le carcinome basocellulaire - L'exérèse large reste le traitement de choix. Plusieurs thérapeutiques comme laser CO_2_ et photothérapie dynamique ont été essayées. Nous rapportons l'observation de Mme G.I âgée de 30 ans présentant une plaque rétro-auriculaire bien limitée évoluant depuis 20 ans. L'examen objective une plaque verruqueuse brunâtre rétro-auriculaire mesurant 3cm de diamètre. Le reste de l'examen est sans particularité. Une kératose séborrhéique, une verrue et un hamartome de Jadassohn ont été évoqués. L'histologie a permis de confirmé le diagnostic d'hamartome de jadassohn. Une exérèse complète a été réalisée. Aucune récidive n'a été notée, le recul est de 3 ans.

**Figure 1:**
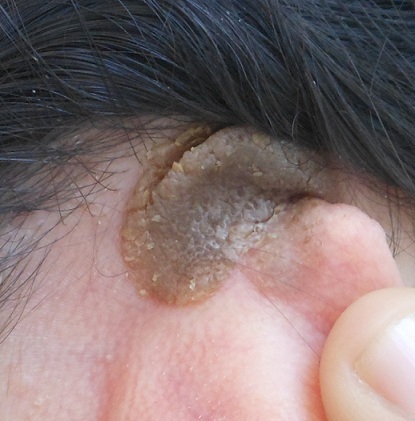
Plaque brun jaunâtre verruqueuse

